# Kinetic Modeling of the Genetic Information Processes in a Minimal Cell

**DOI:** 10.3389/fmolb.2019.00130

**Published:** 2019-11-28

**Authors:** Zane R. Thornburg, Marcelo C. R. Melo, David Bianchi, Troy A. Brier, Cole Crotty, Marian Breuer, Hamilton O. Smith, Clyde A. Hutchison, John I. Glass, Zaida Luthey-Schulten

**Affiliations:** ^1^Department of Chemistry, University of Illinois at Urbana-Champaign, Urbana, IL, United States; ^2^Machine Biology Group, Department of Psychiatry, Microbiology, and Bioengineering, Perelman School of Medicine, University of Pennsylvania, Philadelphia, PA, United States; ^3^Maastricht Centre for Systems Biology (MaCSBio), Maastricht University, Maastricht, Netherlands; ^4^Synthetic Biology and Bioenergy Group, J. Craig Venter Institute, La Jolla, CA, United States

**Keywords:** minimal cells, stochastic simulations, kinetic parameters, DNA replication, transcription, translation, mRNA production, protein production

## Abstract

JCVI-syn3A is a minimal bacterial cell with a 543 kbp genome consisting of 493 genes. For this slow growing minimal cell with a 105 min doubling time, we recently established the essential metabolism including the transport of required nutrients from the environment, the gene map, and genome-wide proteomics. Of the 452 protein-coding genes, 143 are assigned to metabolism and 212 are assigned to genetic information processing. Using genome-wide proteomics and experimentally measured kinetic parameters from the literature we present here kinetic models for the genetic information processes of DNA replication, replication initiation, transcription, and translation which are solved stochastically and averaged over 1,000 replicates/cells. The model predicts the time required for replication initiation and DNA replication to be 8 and 50 min on average respectively and the number of proteins and ribosomal components to be approximately doubled in a cell cycle. The model of genetic information processing when combined with the essential metabolic and cell growth networks will provide a powerful platform for studying the fundamental principles of life.

## 1. Introduction

**Figure 1:**
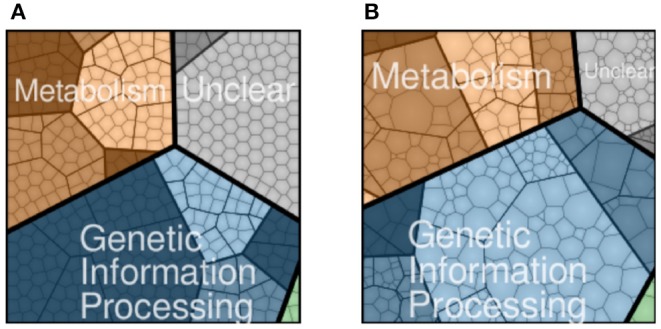
JCVI-syn3A protein coding genes **(A)** and proteomics **(B)** distributed to four functional classes: metabolism (brown), genetic information processing (blue), cellular processes such as cell division (green), and unclear function(gray). Shading within the four functional classes indicate subsystems within the class, such as nucleotide metabolism in metabolism or transcription in genetic information processing. NCBI GenBank CP016816.2: https://www.ncbi.nlm.nih.gov/nuccore/CP016816.2 (Breuer et al., 2019).

JCVI-syn3A, a bacterial cell with a synthetic minimal genome of size 543 kbp and 493 genes, is an organism designed to have the fewest genes necessary for life and is therefore an ideal model organism for studying fundamental principles of life (Lachance et al., 2019). In Breuer et al. (2019), we published the flux balance analysis of the essential metabolism of JCVI-syn3A along with the gene map and the genome-wide data from essentiality and proteomics experiments. Although metabolism, including transport of nutrients into the cell, has been established, the reactions and kinetic models for genetic information processes in JCVI-syn3A are missing. The accompanying gene map in Figure 1A assigned all 452 protein coding genes to one of the four major functional classes: metabolism with transporters (143), genetic information processes (212), cellular processes such as cell division (6), and unclear functions (91). Accompanying the gene map is a map of the proteomics data detected for the 428 proteins in Figure 1B. The model presented here uses the proteomics data to guide the modeling of protein production.

In our previous work on ribosome biogenesis in *Escherichia coli* (Earnest et al., 2015, 2016), ribosome assembly was included along with DNA replication and transcription/translation of just the ribosomal proteins (rproteins). In this simplified model we focus on developing kinetic parameters that replicate the DNA, generate proteins comparable to the proteomics abundances, and produce sufficient numbers of rprotein and ribosomal RNA (rRNA) to generate approximately 500–700 ribosomes estimated from the biomass equation in Breuer et al. (2019). Here we introduce the construction and results of our simplified genetic information processing model for a cell 400 nm in diameter. The kinetics for initiation of DNA replication is based on a mechanism derived from the JCVI-syn3A genomic sequence, crystal structures of the initiator protein DnaA complexed with DNA and kinetics parameters from single molecule fluorescence resonance energy transfer (smFRET) experiments. Parameters for simplified kinetics describing DNA replication, transcription, mRNA degradation, translation, and protein degradation are derived from the literature and our previous studies on JCVI-syn3A (Breuer et al., 2019) and *E. coli* (Earnest et al., 2015, 2016). Within the cell cycle of 105 min, these processes duplicate the genome, generate, and translate sufficient amounts of mRNA to approximately reproduce the proteomics data, and the estimated number of ribosomes. All 452 protein coding genes and 35 genes for rRNAs and tRNAs in the genome of JCVI-syn3A are expressed. Three pseudo genes and three genes for small RNA are not expressed in this model.

## 2. Methods

Each of the genetic information processing subsystems involve species that are low in population in the cell, for example one or two copies of a gene and 0–10 copies of a protein-coding mRNA. To capture the stochastic nature of genetic information processes, the kinetics were modeled with chemical master equation (CME) simulations and solved using the Gillespie algorithm as implemented in the software Lattice Microbes (Roberts et al., 2013; Hallock et al., 2014; Earnest et al., 2015, 2018) with the pyLM interface in a Python 3 Jupyter notebook. Due to the small size of JCVI-syn3A, 400 nm in diameter, we neglect the spatial location of species inside the cell in this simplified model which allows us to stochastically model the kinetics as well-stirred using CME simulations. The results of stochastic simulations were averaged over 1,000 replicates/cells. Each replicate requires a run time of one second. The Jupyter notebooks are available and are posted at GitHub (https://github.com/zanert2/Thornburg_FrontMolBiosci_2019).

### 2.1. Polymerization Model and Rate Forms

In our genetic information processing model, DNA replication, transcription, and translation are all reactions that involve an enzyme (DNAP, RNAP, or ribosome) catalyzing polymerization reactions based on a preexisting template polymer (the entire ssDNA, each unique gene on the ssDNA, or its corresponding mRNA). In the case of replication, the single template is the entire genomic sequence of 543 kpb. In the case of transcription, the templates are the individual 493 genes, each with a unique length and sequence. In the case of translation, the templates are the number of individual messengers for each of the proteins. We use a rate form based on Equation (33) from Hofmeyr et al. (2013) that was derived assuming polymerization from a single unique template where the enzyme is in excess and the concentration of free enzyme is constant. DNA replication, transcription, and translation all involve a situation in which the enzyme is in excess of unique templates. For DNA replication, there is a single start site, oriC, and 35 DNAP molecules in the proteomics data. In the case of transcription, there are 187 RNAP and if we consider any one gene as the template for the rate form, there are at most two copies of the gene at any point in the cell cycle. In translation, there are over 500 ribosomes available to translate the individual mRNAs which typically number <10. In each case, we assume a constant steady-state concentration of free enzymes in determining the kinetic rates, although the template concentrations will change over time. The general polymerization rate form can be written as

(1) $$\begin{array}{l} v_{poly}=\frac{k_{cat}\left[T\right]}{\left(1+\frac{K_{0}}{\left[E\right]}\right)\frac{K_{D1}K_{D2}}{{\left[M\right]}_{1}{\left[M\right]}_{2}}+\sum_{i}\frac{n_{i}K_{Di}}{{\left[M\right]}_{i}}+n_{tot}} \end{array}$$

which we modify for transcription and translation in the following sections to address that there is competition among unique templates of different lengths *n*_*tot*_ in each process. For our experimental situation, the polymerization rate is dominated by *k*_*cat*_, *n*_*tot*_, and template concentrations. The variation in rates based on these assumptions is discussed further below in Equation (2). The general rate form considers a mechanism starting with enzyme *E* (DNAP, RNAP, or ribosome) binding to a polymer template *T* with binding constant *K*_0_. Once the enzyme and template have bound, the first two monomers (dNTP, NTP, or the charged aa-tRNA) *M*_1_ and *M*_2_ bind to the template/enzyme complex with association constants *K*_*D*1_ and *K*_*D*2_. The monomer concentrations are determined by the pool sizes provided in Zhang and Ignatova (2009) and Breuer et al. (2019). A value of *K*_*D*_ has been measured for a single elongation step of mRNA by RNAP, but not for DNAP or ribosomes (Larson et al., 2012). Values for *K*_*D*_ were fitted to maximize the rate of each process assuming their respective pool sizes and other experimentally measured kinetic parameters. Our fitted value for RNAP agrees well with the experimentally determined value. Monomers of type *i* are then added to the growing polymer by the binding with their respective association constant *K*_*Di*_ and we assume that they are the same for any one process. The growing polymer is elongated at a rate *k*_*cat*_. The resulting polymer (DNA, rRNA, mRNA, tRNA, or protein) of length *n*_*tot*_ will consist of *n*_*i*_ of each respective monomer type *M*_*i*_ following the first two positions in the polymer.

In general, both the enzyme and template concentrations are functions of time. In evaluating the rate constant, the enzyme concentrations were held constant to the values derived from the proteomics data making the polymerization rate obey first order kinetics

(2) $$\begin{array}{l} \nu =k\left(n_{tot},k_{cat}\right)\left[T\right] \end{array}$$

where the rate constant is defined as

(3) $$\begin{array}{l} k\left(n_{tot},k_{cat}\right)=C\times \frac{k_{cat}}{\left(1+\frac{K_{0}}{\left[E\right]}\right)\frac{K_{D1}K_{D2}}{{\left[M\right]}_{1}{\left[M\right]}_{2}}+\sum_{i}\frac{n_{i}K_{Di}}{{\left[M\right]}_{i}}+n_{tot}} \end{array}$$

in which *C* represents any modifications to the rates of transcription or translation. For the kinetic parameters, pool sizes, and low enzyme concentrations assumed in the kinetic model, the denominator is dominated by the third term, the length of the new polymer *n*_*tot*_. In analyzing the sensitivity of DNA replication, transcription, and translation to the concentration of each respective enzyme, we found that the rate constants *k* from Equation (3) deviated no more than 10^−4^% as the concentration of enzyme is doubled over the cell cycle. Our above approximations hold assuming the cell is in the exponential growth phase where nutrient and pool sizes are in a steady state. The approximations no longer hold in cases such as the transition from exponential to stationary growth. As nutrients in the environment become depleted, the rate of elongation steps in DNA replication, transcription, and translation will be slowed down due to a lack of monomers *M*_*i*_.

### 2.2. Replication Initiation

**Figure 2:**
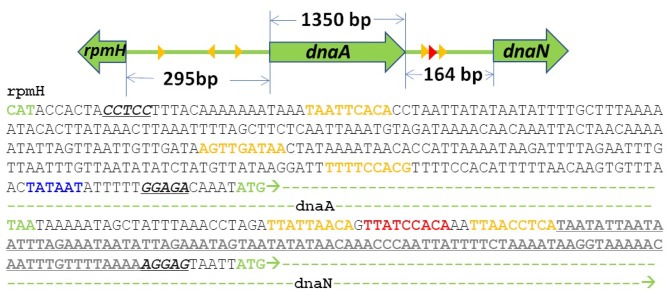
DNA sequence near oriC of JCVI-syn3A defined by the 3 high (red) and low (yellow) affinity DnaA(IV) binding sites: The AT-rich region (bold and underlined) binds DnaA(III). The AT-rich region ends at the Shine-Dalgarno sequence (italicized and underlined) preceding the dnaN gene (green dashes). A putative promoter for the DnaA gene preceding its Shine Dalgarno sequence is shown in blue.

**Figure 3:**
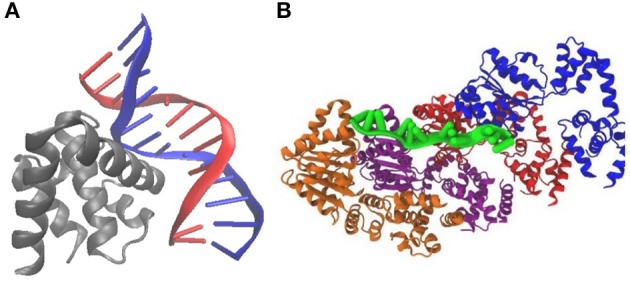
Crystal structures of DnaA binding to *E. coli* DNA suggest a mechanism for initiation of replication: **(A)** PDB 1J1V; DnaA(IV) binds to a 9-bp signature on dsDNA. **(B)** PDB 3R8F; Four DnaA(III) bind to 3-nucleotide increments on ssDNA.

Previous treatments of replication initiation have proposed a mechanism based on *E. coli* and *B. subtillis* that began with the initiator protein DnaA binding to four 9-bp signatures of the DNA near oriC, followed by accumulation of DnaA monomers around that location until a buildup of 20–30 monomers was reached (Atlas et al., 2008; Karr et al., 2012). Our model of DNA replication initiation is based on the genomic sequence of JCVI-syn3A in Figure 2 and a mechanism derived from crystal structures of the multi-domain DnaA binding to ds- and ssDNA shown in Figure 3. In the genomic sequence structure, a strong DnaA binding signature (TTATCCACA) is located near the origin matching the whole 9-bp sequence with two neighboring signatures matching 7 out of 9 bp (Schaper and Messer, 1995; Weigel et al., 1997; Speck et al., 1999). These signatures lie next to an AT-rich region 93 bp in length.

**Figure 4:**
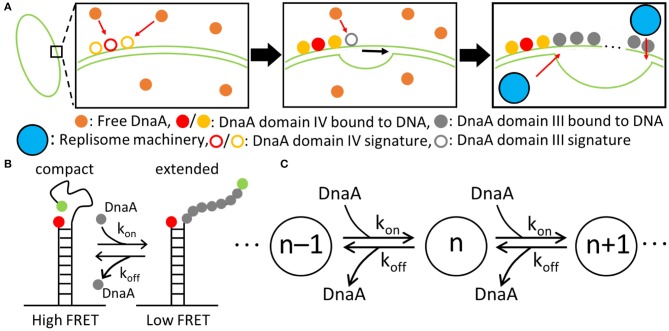
Replication initiation mechanism. **(A)** DnaA(IV) binds to three signatures on dsDNA next to the AT-rich region near oriC. DnaA(III) subsequently binds in 3-nucleotide increments. DnaA(III) continues to bind to ssDNA until the AT-rich region is opened, allowing the replisome machinery to be loaded. **(B)** The kinetic parameters for the binding of DnaA(III) to ssDNA were obtained from a smFRET study (Cheng et al., 2014) where the FRET signal depended on the number of DnaA bound. Fewer DnaA corresponded to compact ssDNA, resulting in a high FRET signal. Increasing the number of DnaA bound to ssDNA extends the filament, lowering the FRET signal. **(C)** Schematic of the binding kinetics of DnaA(III) to ssDNA forming a DnaA filament of length n. The *k*_*on*_ and *k*_*off*_ values correspond to the kinetics measured by smFRET.

DnaA domain IV [DnaA(IV)] binds most strongly to the sequence TTATCCACA. DnaA(IV) binds to the dsDNA signatures (Erzberger et al., 2006; Duderstadt et al., 2011). DnaA domain III [DnaA(III)] binds to AT-rich ssDNA in 3 nucleotide increments forming a helical, filament-like structure (Erzberger et al., 2006; Duderstadt et al., 2011). Our mechanism assumes that the binding of DnaA(IV) to the three neighboring dsDNA signatures near oriC opens up a small pocket of ssDNA in the neighboring AT-rich region. This mechanism is illustrated in Figure 4A. Once the dsDNA sites are occupied, DnaA(III) can start binding to the neighboring AT-rich region on the ssDNA. The DNA continues to be unwound until the AT-rich region is wrapped by the DnaA filament. Since DnaA(III) binds to ssDNA in 3 nt increments (Duderstadt et al., 2011; Cheng et al., 2014) the 93 bp AT-rich region shown in Figure 2, produces a filament with 30 DnaA. After formation of the filament, replication can be initiated.

To capture the proposed mechanism, we begin with a reaction binding a DnaA to the high affinity binding signature near OriC on dsDNA, creating a bound site and the two low affinity free sites on either side of the high affinity site. The low affinity sites on dsDNA then react with one DnaA each, creating a bound site for each. The dsDNA binding rates use second order rate forms using the rate constants shown in Table 1. There is also a reaction in the model for DnaA binding to other high affinity sites around the chromosome. This is included since the filament length strongly depends on the number of free DnaA available. The kinetic model for the formation of the DnaA filament is based on an smFRET study on ssDNA (Cheng et al., 2014). The smFRET study in Figure 4B reports values for *k*_*on*_ for addition of a DnaA molecule to the growing DnaA filament bound to ssDNA and *k*_*off*_ for removal of a DnaA molecule from the filament as shown in Figure 4C. These kinetic parameters are presented in Table 1 and were used for each independent binding and unbinding until a filament consisting of 30 DnaA has formed. Once the filament is formed and replication begins, the filament is assumed to be removed at the rate of the polymerization in DNA replication which models removal of DnaA by DNA helicase. The model is constructed so that only one replication initiation event occurs in a cell cycle.

**Table 1:** Kinetic parameters used in the model of replication initiation.

**Parameter**	**Value**	**Units**	**References**
High affinity binding rate	7,800	mM^−1^ s^−1^	Schaper and Messer, 1995; Weigel et al., 1997
Low affinity binding rate	35	mM^−1^ s^−1^	Schaper and Messer, 1995; Weigel et al., 1997
*k*_*on*_	100	mM^−1^ s^−1^	Cheng et al., 2014
*k*_*off*_	0.55	s^−1^	Cheng et al., 2014

### 2.3. Replication

**Figure 5:**
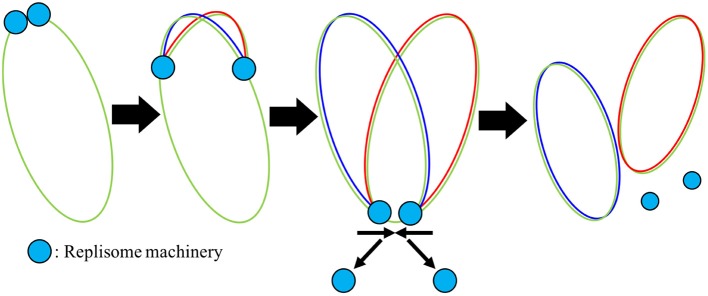
Mechanism of DNA replication: Once the replisome machinery is loaded onto the chromosome shown in green, the machinery begins to polymerize around the DNA, elongating new DNA onto both halves of the original chromosome shown in red and blue. Once the replisome reaches the terminus, we assume that the replisomes fall off quickly.

The replisome, a complex containing proteins necessary for DNA replication including DNA helicase, DNAP, DNA primase, gyrase/topoisomerase, and the beta clamp, binds at oriC once the replication initiation event has occurred and then proceeds in both directions around the chromosome, creating the two replication forks as shown in Figure 5. Using smFRET experiments, the replisome has been observed to assemble in just a few seconds (Downey and McHenry, 2010; Cho et al., 2014). We do not model the assembly of the replisome and assume its assembly occurs during or before replication initiation. As the replisome proceeds along the chromosome, the original chromosome shown in green is unzipped and the two new chromosomes shown in red and blue are polymerized on the original ssDNA template. Both strands of ssDNA at the replication fork are treated the same with continuous polymerization, and okazaki fragments are not modeled. The model assumes that once the replisomes reach the terminus, they fall off quickly and the two new chromosomes are instantaneously separated. The number of dATP, dTTP, dCTP, and dGTP monomers *n*_*i*_ appearing in the rate form (Equation 1) are calculated from the A, T, C, and G content of the genome: 203606 A, 207816 T, 67238 C, and 64720 G. Since there are no metabolic reactions to produce deoxynucleotides or ATP for the reactions to occur, constant pools for each are assumed using the pool sizes from Breuer et al. (2019) presented in Table 2.

**Table 2:** Pool sizes from Breuer et al. (2019) and estimated from Zhang and Ignatova (2009) and Mackie (2013)^*^.

**Species**	**Pool size (mM)**
dATP	0.018
dTTP	0.022
dCTP	0.012
dGTP	0.007
ATP	1.04
UTP	0.68
CTP	0.34
GTP	0.68
tRNA^*^	0.0020
aa-tRNA^*^	0.0076

Kinetic parameters for replication are given in Table 3. The elongation rate constant *k*_*cat*_ (Xie et al., 2008) and the association constant for DNAP to DNA *K*_0_ (Zhang et al., 2016) were obtained from the literature for *E. coli*. In order to make a second copy of the genome within the 105 min doubling time, the choice of *K*_*D*_ was made in order to minimize the time to duplicate the DNA. Assuming the constant pool sizes and DNAP concentrations, the value of *K*_*D*_ corresponds to the value where the length of the genome is the dominant term in the denominator of *k* in Equation (3).

**Table 3:** Parameters used in kinetics for replication, transcription, translation, mRNA degradation, and protein degradation.

**Subsystem**	**Parameter**	**Value**	**Units**	**References**
Replication	*k*_*cat*_	600	bp/s	Breier et al., 2005; Xie et al., 2008
	*K*_0_	0.26	μM	Zhang et al., 2016
	*K*_*D*_	1.0	μM	Fitted
Transcription	*k*_*cat*_ (mRNA and tRNA)	25	nt/s	Chen et al., 2015
	*k*_*cat*_ (rRNA)	180	nt/s	Ryals et al., 1982
	*K*_0_	100	nM	Bremer and Dennis, 2008
	*K*_*D*_	0.1	mM	Fitted; Larson et al., 2012
Translation	*k*_*cat*_	5	aa/s	Cox, 2004
	*K*_0_	100	nM	Bremer and Dennis, 2008
	*K*_*D*_	0.01	mM	Fitted
mRNA Degradation	*t*_1/2_	4	min	Bernstein et al., 2004; Briani et al., 2008
Protein degradation	*t*_1/2_	25	hr	Maier et al., 2011

### 2.4. Transcription

To modify the general rate form for transcription, we incorporate two factors: the probability of an active RNAP selecting any gene *P*_*gene selection*_ and the strength of the gene's promoter *S*_*promoter*_. The fraction of active RNAP as estimated by Bremer and Dennis (2008) for a cell with a ~100 min doubling time implies that around 29 of the 187 RNAP are actively transcribing at any time. Of the actively transcribing RNAPs, Bremer and Dennis (2008) estimate that approximately 24% are involved in making stable RNA like rRNA. Since each rRNA operon only contains the 16S, 23S, and 5S rRNAs and no tRNAs, transcription of the two drRNA genes will require four RNAP. Therefore, the probability of any other gene being selected is *P*_*gene selection*_ = 25/487 = 0.05. We estimate that each rRNA operon is always being actively transcribed by two RNAP, and therefore has a probability of gene selection of 1. The expression from Hofmeyr et al. (2013) did not include competition for multiple templates which is now captured with the probability of gene selection. This gives us a transcription rate

(4) $$\begin{array}{l} \nu_{transcription}=P_{gene\;selection}\times \nu_{poly} \end{array}$$

which we use for transcription of rRNA, tRNA, and ribosomal protein-coding genes.

The the rate of transcribing a gene also depends on the strength of its promoter sequence (Jones et al., 2014), however the precise promoter sequences and their strengths have not been measured for JCVI-syn3A. In a preliminary analysis of the sequences preceding each protein-coding gene, we found that, in general, a protein is more likely to have a higher proteomics value if the start codon is preceded by both a Shine Dalgarno sequence a promoter sequence TANAAT as characterized in *Mycoplasma pneumoniae* (Lloréns-Rico et al., 2015). Using this information, to incorporate a proxy for promoter strength, *S*_*promoter*_, into the kinetics, the transcription rate for each non-ribosomal protein coding gene is multiplied by the ratio of gene's proteomics count to the average proteomics count of 180

(5) $$\begin{array}{l} \nu_{mRNA\;transcription}=S_{promoter}\times P_{gene\;selection}\times \nu_{poly} \end{array}$$

Since some ribosomal proteins were not reported in the proteomics data, this factor is not used in the transcription rates of ribosomal protein coding genes.

The model expresses the genes for all 452 protein coding genes and the genes for rRNA and tRNA. For each protein or RNA, the gene identifier from the NCBI entry (NCBI GenBank CP016816.2: https://www.ncbi.nlm.nih.gov/nuccore/CP016816.2; Breuer et al., 2019) is read and the corresponding sequence is used to determine the nucleotide stoichiometries for the formation and degradation reactions. RNA formation reactions use our modified polymerized, template-driven rate forms in Equations (4) and (5) and the degradation reactions of mRNA follow first order kinetics. The nucleotide stoichiometries are used to determine the monomer counts *n*_*i*_ and total polymer length *n*_*tot*_ in the rate form. Constant pools of nucleotides are assumed using the pool sizes from Breuer et al. (2019) presented in Table 2. For the transcription reactions, the enzyme is RNAP and the template is the total concentration of the gene in the cell as a function of time and includes the replication of DNA. This model, however, does not take into account the location of a gene on the genome during DNA elongation. The elongation rate constant *k*_*cat*_ and the association constants *K*_0_ and *K*_*D*_ are listed in Table 3. Literature values of mRNA and tRNA elongation rates of 25 nt/s are used for *k*_*cat*_ (Chen et al., 2015). A messenger half-life of 4 min is used for all mRNA degradation. The half-life of 1 min in Breuer et al. (2019) did not result in mRNA abundances that produced proteins quickly enough to double the number of proteins in the cell cycle. The 4 min half life gives a total mRNA abundance in better agreement with the data published in Lynch and Marinov (2015). The experimentally observed rRNA operon elongation rate *k*_*cat*_ of 90 nt/s (Ryals et al., 1982) was multiplied by two for both operons to model the effect of two RNAP simultaneously transcribing each operon. The association constant for association of RNAP to DNA *K*_0_ was calculated according to Hofmeyr et al. (2013) using the concentrations of the free and actively transcribing RNAP (Bremer and Dennis, 2008) and concentration of the gene. The association constant for nucleotides binding to the RNAP/gene complex *K*_*D*_ was fitted so that the rate of transcription was maximized by making transcript length the dominant term in the denominator of *k* in Equation (3). Our fitted value agrees with a measured experimental value of 0.14 mM (Larson et al., 2012). With no transcriptomic data available, each mRNA begins with a count of 1 and each tRNA is divided evenly at 190 each to have a total tRNA abundance of 3,750, a value scaled from *E. coli* based on differences in cell volume (Mackie, 2013).

### 2.5. Translation

Since the number of total mRNA is approximately on the same order of the number of ribosomes, the probability of any mRNA being translated is near unity. The only other modification of the translation rate expression is to allow more than one ribosome (polysomes) *N*_*ribo*_ to bind to a long transcript in Equation (6). This factor is an integer calculated as the length of the transcript over an estimated ribosome spacing of 300 nt in *E. coli* (Brandt et al., 2009). If the value is calculated as <1, the value of *N*_*ribo*_ is set to 1. The ribosome spacing was estimated using an observed approximate average of 4 ribosomes per polysome for an average transcript length of 1,200 nt.

(6) $$\begin{array}{l} \nu_{translation}=N_{ribo}\times \nu_{poly} \end{array}$$

The model includes the translation and degradation of each protein made from each mRNA. The gene identifier from the NCBI entry also includes the amino acid sequence for protein coding genes which is used to determine the corresponding stoichiometries of tRNA charged with their corresponding amino acids (aa-tRNA) required to build the protein and the amino acid stoichiometries when the protein is degraded. For the translation reactions, the template in the polymerization rate form (Equation 1) is the associated mRNA. The model uses whole, intact ribosomes as the enzyme and does not model association of messengers to the 30S small subunit followed by association of the 50S large subunit. The elongation rate constant *k*_*cat*_ and the association constants *K*_0_ and *K*_*D*_ are listed in Table 3. For *E. coli*, experimentally measured elongation rates range from 10 to 20 aa/sec (Bremer and Dennis, 2008), however slower rates have been reported in other bacteria such as *Mycobacterium bovis* with an elongation rate of 2 aa/sec (Cox, 2004). A value within the estimated range of 2–10 aa/sec of 5 aa/sec was chosen so that the number of proteins was approximately doubled in a cell cycle. The association constant of the ribosome to the mRNA *K*_0_ was estimated using the average fraction of actively translating ribosomes (Bremer and Dennis, 2008) and an average concentration of an mRNA to be one in the cell. The association constant for aa-tRNA binding to the ribosome/mRNA complex *K*_*D*_ was fitted to maximize the rate of translation assuming constant aa-tRNA pool sizes and ribosome concentration. The value of *K*_*D*_ was computed using the length of the shortest protein, ribosomal protein L34 (40 aa), in the equation for the rate constant *k* (Equation 3). A half-life of 25 h was used for protein degradation reactions (Maier et al., 2011) Degradation of the proteins in extremely slow, so the main source of dilution would be by cell division after 105 min.

### 2.6. ATP Energy Costs

Replication, transcription, translation, mRNA degradation, and protein degradation have associated ATP hydrolysis costs. Although the mechanism for ATP hydrolysis is not explicitly modeled, the costs are incorporated as additional time dependent reactions for each subsystem. For example, in DNA replication the DNA helicase is not explicitly modeled, but we assume that 1 ATP hydrolysis event per bp is required to unwind the dsDNA. The ATP cost of each reaction in each subsystem is determined by the length of the DNA/RNA/protein being formed or mRNA/protein being degraded (Russell and Cook, 1995; Lynch and Marinov, 2015). In transcription, we assume that the RNAP uses 1 ATP hydrolysis event per bp to unwind the dsDNA. The mRNA degradation reactions also assume that 1 ATP hydrolysis event is required per nucleotide removed from the messenger. The transcription reactions assume 2 ATP hydrolysis events per amino acid addition. These reactions use 2 instead of 4 ATP hydrolysis events since the amino acid charging of the tRNA are already included in the essential metabolic network (Breuer et al., 2019). The costs used are also shown in Table 4.

**Table 4:** ATP hydrolysis costs of reactions in genetic information processing subsystems (Russell and Cook, 1995; Lynch and Marinov, 2015).

**Reaction**	**ATP cost**	**Units**
Replication	1	ATP per bp
Transcription	1	ATP per nt
Translation	2	ATP per aa
mRNA degradation	1	ATP per nt
Protein degradation	1	ATP per aa

*The cost of translation does not include charging of the tRNAs as those reactions are incorporated in the essential metabolism (Breuer et al., 2019)*.

## 3. Results

### 3.1. Replication Initiation and Replication

**Figure 6:**
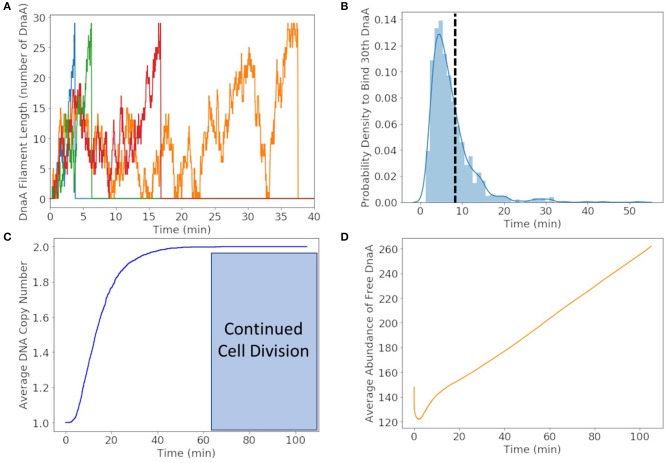
**(A)** DnaA filament formation for four different replicates shown in different colors. The stochastic effects of the filamentation kinetics result in a wide range of times to form the filament from <5 to 50 min. **(B)** Probability distribution of replication initiation times when the thirtieth DnaA in the ssDNA filament binds. We predict the most probable time to form the filament to be approximately 5 min and the average time to be approximately 8 min shown with a dotted line. **(C)** Average of genome duplication over 1,000 replicates shows that on average the genome will be duplicated in 65 min of the 105 min cell cycle, leaving approximately 40 min for continued cell division. **(D)** The average abundance of DnaA not bound to DNA gets depleted by filament formation and replenished by translation and removal of the filament by DNA helicase.

We found that DnaA(IV) requires <1 min to bind to all three dsDNA signatures. The stochastic trajectories of DnaA filament formation from four representative cells are shown in Figure 6A. The distribution of times to form the DnaA filament in Figure 6B is peaked at 5 min, but on average it takes 8 min for the DnaA filament to form on ssDNA as shown with a dotted line. Once the filament is 30 DnaA in length, replication begins and the DnaA filament is removed by the polymerization of DNA, resulting in the fast drop from 30 to 0 DnaA in the filament as seen in the trajectories in Figure 6A. It then takes another 50 min on average for replication to reach completion in Figure 6C. We predict replication initiation and replication are completed by 65 min, leaving another 40 min for the cell to divide in the 105 min cell cycle.

To illustrate the time-dependent variation in protein formation, the average abundance of free DnaA is shown in Figure 6D. Within the first minute we see a fast drop due to DnaA(IV) binding to high affinity dsDNA binding sites around the genome. The filament formation slowly removes DnaA from the free DnaA abundance until around 8 min when replication most frequently begins. DnaA in then replenished over several minutes due to removal of the filament by DNA helicase and translation of new DnaA.

### 3.2. Transcription

**Figure 7:**
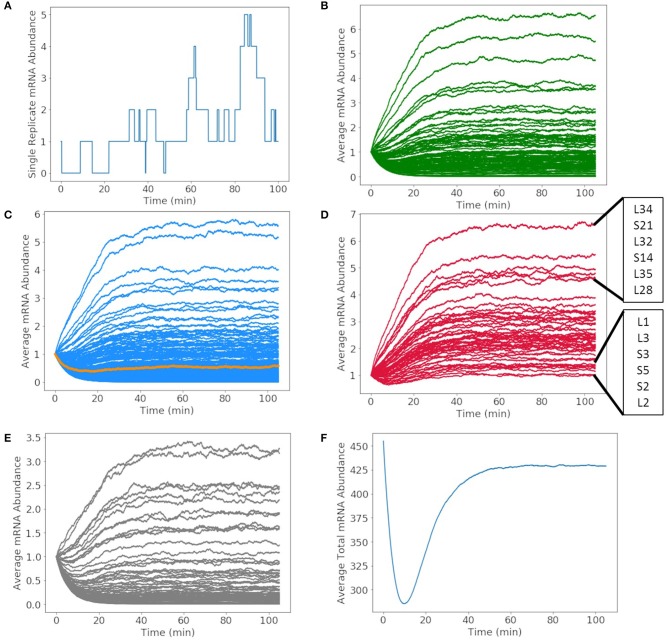
Abundances of mRNA and tRNA transcribed in a 105 min cell cycle. **(A)** A single replicate from the stochastic simulation of the mRNA abundance for glucose-6-phosphate isomerase shows fluctuations in the average integer abundance of messengers. Fluctuations arise from competing rates of formation, degradation, and replication. The average mRNA abundances of mRNA coding for **(B)** metabolic proteins, **(C)** genetic information processing, DnaA (orange), and cell division proteins, **(D)** ribosomal proteins, and **(E)** proteins of unclear function all have average abundances between zero and seven. **(F)** The total number of all messengers during a cell cycle averaged over 1,000 replicates shows that typically there are 300–450 messengers present in the cell at any time.

The mRNA production in a single cell exhibits fluctuations due to competing rates of formation and degradation. A representative of the mRNA production for glucose-6-phosphate isomerase over the 105 min simulation is shown in Figure 7A. The abundance of the messenger fluctuates from zero to two before DNA replication occurs and then one to five once the gene has been duplicated. The time dependence of all mRNA over a cell cycle averaged over 1,000 replicates are shown in Figures 7B–E. The mRNA are divided by mRNA for metabolic proteins (Figure 7B), genetic information processing and cell division proteins (Figure 7C), ribosomal proteins (Figure 7D), and proteins of unclear function (Figure 7E). The resulting kinetics show each mRNA growing or depleting in population from the initial one copy until the effects of replication are fully manifested around 60 min. In the early phase, the increase or decrease of mRNA reflects the competition between mRNA decay and the length of the transcript and the strength of the gene's promoter. As the genome is duplicated, this equilibrium for each mRNA shifts once a second copy of the gene is present. As the position of the gene in the genome is not considered, the variations are proportional to change in the DNA copy number of the cell cycle and not the nearness to oriC. The total number of mRNAs in Figure 7F varies from its initial value of 452 (one for each of the protein-coding genes) to an equilibrium value of approximately 425.

**Figure 8:**
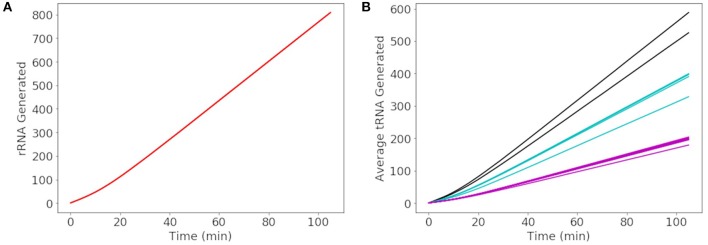
Generated abundances of rRNA and tRNA for a cell cycle averaged over 1000 replicates. **(A)** 16S, 23S, and 5S rRNA are each generated to the same average abundance of 800 over a cell cycle. **(B)** The average abundance of each tRNA generated in a cell cycle results in a total abundance of approximately 4000 tRNAs. The tRNAs in black are methionine and leucine, in blue are threonine, tryptophan, lysine, arginine, and serine, and remaining tRNAs are shown in pink.

More than 500 of each rRNA were produced in a cell cycle shown in Figure 8A, reaching the number required to produce 500–700 ribosomes in the cell cycle estimated by Breuer et al. (2019). The number of each tRNA produced in Figure 8B reveals three groupings of tRNA production. The three groupings depend on the number of genes for each tRNA present in the genome. The groups consisting of more than one gene include 3 each of methionine and leucine tRNA genes making up the tRNA grouped between 500 and 600 tRNA and 2 each of threonine, tryptophan, lysine, arginine, and serine tRNA genes making up the tRNA grouped between 300 and 400 tRNA. Overall the model produces approximately 4,000 total tRNAs over a cell cycle, in close agreement with the initial estimate of 3,750 obtained from scaling the abundances in *E. coli* (Mackie, 2013).

### 3.3. Translation

**Figure 9:**
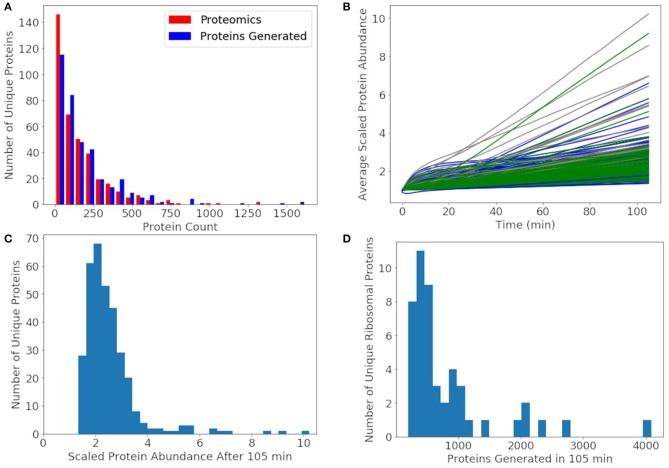
**(A)** The distribution of proteins generated after 105 min averaged over 1,000 replicates approximately reproduces the experimental proteomics distribution for JCVI-syn3A. **(B)** Average protein counts scaled to the proteomics numbers for proteins with a proteomics counts >10. Metabolic proteins (green), non-ribosomal genetic information processing and cell division proteins (blue), and proteins of unclear function (gray). **(C)** A histogram of the scaled protein abundances at 105 min shows that the model doubles the abundances of most proteins with only a few outliers including mostly proteins of unclear function and thioredoxin, acyl carrier protein, transcription antitermination factor NusB, aspartyl/glutamyl-tRNA amidotransferase, and transporter ptsH. **(D)** A histogram of the number of ribosomal proteins generated shows that the model produces approximately 500 of most ribosomal proteins, enough to form the predicted 500 ribosomes. Some ribosomal proteins were produced in large excess including L34 above 4000, S21 near 3000, and L32, L35, S14, and L28 around 2000 each.

Since the protein degradation rate of 25 h is much slower than the mRNA degradation rate of 4 min, proteins will accumulate and only decay significantly by dilution through cell division. The goal of the model was to approximately reproduce the experimental proteomics distribution, double the abundance of each non-ribosomal protein, and produce 500–700 of each ribosomal protein. We compare our distribution of generated proteins over a cell cycle to the experimental proteomics in Figure 9A. We approximately reproduce most of the distribution with the greatest deviation being for proteins with fewer than 10 counts in the proteomics data. In the rest of our analysis of non-ribosomal proteins, we focus on proteins with experimental proteomics abundances >10. For further comparison, the number of each non-ribosomal protein generated over a cell cycle is compared to its proteomics value used to initialize the simulations (Figure 9B). From the histogram in Figure 9C we see that most non-ribosomal proteins double in number over a cell cycle with a few outliers, of which most are proteins of unclear function. The remaining outliers include thioredoxin, acyl carrier protein, transcription antitermination factor NusB, aspartyl/glutamyl-tRNA amidotransferase, and ptsH, all of which are short proteins around 100 amino acids in length or shorter. The histogram of ribosomal proteins abundances generated by the model in Figure 9D reveals that the model produces 500 copies for the majority of the ribosomal proteins, while the shortest are being overproduced. Ribosomal proteins overproduced include L34 above 4,000, S21 near 3,000, and L32, L35, S14, and L28 above 2,000 each. Ribosomal proteins not generated to an abundance of at least 500 include L1, L3, S3, S5, S2, and L2.

### 3.4. ATP Energy Costs

The model was constructed to estimate the ATP hydrolysis requirements for the genetic information processes in the minimal cell using per bp, nt, or aa usage of ATP in DNA elongation, transcription, translation, mRNA degradation, and protein degradation. The estimates of the ATP hydrolysis cost over a 105 min simulation are presented in Table 5 as both the total number of ATP used and the corresponding concentration of ATP required for a 400 nm cell. The model predicts that the total ATP hydrolysis cost over a cell cycle to be approximately 3,800 mM for JCVI-syn3A. This estimate does not suggest that 3,800 mM of ATP needs to be present in the cell, but provides an estimate for how quickly the metabolism will need to convert ADP into ATP. The most significant of the ATP hydrolysis costs in the genetic information processes comes from translation requiring 2,900 mM and the smallest of the costs is for DNA replication at 28 mM. The cost for translation will be higher once the genetic information processes are paired with the metabolism, as this cost did not account for the two ATP hydrolysis events to charge each tRNA which are included in the essential metabolism (Breuer et al., 2019). The cost for transcription of 500 mM does not include the ATP built into RNA sequences, it only includes the ATP hydrolysis costs of the RNAP. The predicts ATP requirements for mRNA degradation and protein degradation are predicted to be 290 and 90 mM, respectively. The cost for protein degradation is smaller due to the long protein have-life of 25 h relative to the 4 min half-life of messengers.

**Table 5:** ATP hydrolysis costs of the deterministic model for genetic information processes.

**Subsystem**	**ATP used in 105 min (millions)**	**ATP cost for a 400 nm cell (mM)**
Total	77	3,800
Replication	0.54	28
Transcription	10	500
Translation	59	2,900
mRNA degradation	5.9	290
Protein degradation	1.9	93

*The ATP cost for transcription reported here only includes the hydrolysis costs of the RNAP, it does not include ATP built into RNA sequences*.

## 4. Discussion

Our detailed model for the initiation of DNA replication builds upon observations from crystal structures of the initiator protein DnaA bound to signatures on ds-and ssDNA found near the oriC and smFRET measurements of the DnaA filament formation on ssDNA. The time taken for DNA replication initiation is predicted to vary from <5 min up to 50 min. We predict a total time of 65 min on average for the formation of the second copy of the genome, which means at least one copy of the DNA can be generated in a cell cycle.

The average number of any mRNA is within the expected range from zero to ten as reported in *E. coli* (Milo and Phillips, 2015) and can be used as predictions for mRNA counts in JCVI-syn3A until transcriptomic data or smFISH experiments are available for validation. We predict that approximately 450 messengers will be present in the cell on average, agreeing with the extrapolated number for a 400 nm diameter cell from Lynch and Marinov (2015). In our previous treatments of replication and transcription of a given gene in *E. coli* (Peterson et al., 2015; Cole and Luthey-Schulten, 2017) we showed how the variation in DNA copy number and position of the gene in circular DNA can broaden the mRNA distribution. We are likely underestimating the distributions for genes close to oriC and overestimating the distributions for genes near the terminus. In the case of rRNA, a higher transcription rate generated a sufficient number of rRNA to form 500–700 ribosomes in a cell cycle. A higher transcription rate was justified from the greater promoter strength of the rRNA operon observed in *E. coli* and other bacteria (Maeda et al., 2015) as well as the presence of multiple RNAPs estimated to be reading the operon (Bremer and Dennis, 2008). While the model produces over 500 rRNAs, there is variation in the number of ribosomal proteins. For the majority of the ribosomal proteins, approximately 500 of each were generated. However, the long ribosomal proteins were not generated quickly enough and the shorter ribosomal proteins occurred in much higher numbers. This is likely due to no promoter strength being assigned to the transcription of genes coding for ribosomal proteins. In the case of non-ribosomal proteins where we assigned promoter strengths based on proteomics counts, our model, to the most part, approximately doubles the number of proteins over a cell cycle. Identification of the promoter sequences and operonal structures for genes in JCVI-syn3A would help assign variation in promoter strengths and transcription rates on the basis of genomic information rather than proteomics values.

The simplified kinetic models for the genetic information processing reactions in the minimal cell JCVI-syn3A neglected the explicit assembly of the protein complexes that replicate DNA (replisome), transcribe the genes, and translate the mRNA and instead focused on the “polymerization” reactions that replicated the DNA, transcribed the genes into mRNAs, and translated them into proteins and how they are coupled. In some cases, this neglect can be justified by assumed timescale separation of the processes, but in general more experimental measurements of the assembly reactions would help to establish to what degree the association of the complexes are captured in the kinetic parameters given in the literature for the fundamental processes of replication, transcription, and translation. As the next step, the results from the genetic information processes will first be connected to uptake reactions that transport nucleobases, nucleosides, and amino acids into the minimal cell. Coupling genetic information processes with the essential metabolism and cell growth should result in a complete whole cell kinetic model of JCVI-syn3A.

## Data Availability Statement

The jupyter notebooks containing the models in this study can be found at https://github.com/zanert2/Thornburg_FrontMolBiosci_2019.

## Author Contributions

ZT and ZL-S: developed models for genetic information processes, data curation, writing—original draft, and writing—reviewing and editing. MM: assistance in writing of Jupyter notebooks. DB and TB: advised Lattice Microbes interface for the stochastic model. HS: assisted in development of DNA replication initiation model. CC: constructed initial stochastic model of replication initiation. MB: data curation. CH and JG: reviewing.

### Conflict of Interest

The authors declare that the research was conducted in the absence of any commercial or financial relationships that could be construed as a potential conflict of interest.
